# A Study of Mixed Non-Motorized Traffic Flow Characteristics and Capacity Based on Multi-Source Video Data

**DOI:** 10.3390/s24217045

**Published:** 2024-10-31

**Authors:** Guobin Gu, Xin Sun, Benxiao Lou, Xiang Wang, Bingheng Yang, Jianqiu Chen, Dan Zhou, Shiqian Huang, Qingwei Hu, Chun Bao

**Affiliations:** 1 Guangxi Key Laboratory of International Join for China-ASEAN Comprehensive Transportation, Nanning University, Nanning 530200, China; guguobin@unn.edu.cn (G.G.); benxiaolou@csu.edu.cn (B.L.); 15007713136@163.com (C.B.); 2 China Nuclear Engineering Consulting Co., Ltd., Beijing 102200, China; lynn@163.com; 3 School of Traffic and Transportation Engineering, Central South University, Changsha 410075, China; 4 Guangxi Key Laboratory of Intelligent Transportation System (ITS), Guilin University of Electronic Technology, Guilin 541004, China; 17339200188@163.com (X.W.); 15726081096@163.com (B.Y.); 15251707205@163.com (D.Z.); hsq1216640923@outlook.com (S.H.); 199711@163.com (Q.H.)

**Keywords:** traffic flow theory, mixed traffic flow, capacity, stochastic signals, power spectrum estimation

## Abstract

Mixed non-motorized traffic is largely unaffected by motor vehicle congestion, offering high accessibility and convenience, and thus serving as a primary mode of “last-mile” transportation in urban areas. To advance stochastic capacity estimation methods and provide reliable assessments of non-motorized roadway capacity, this study proposes a stochastic capacity estimation model based on power spectral analysis. The model treats discrete traffic flow data as a time-series signal and employs a stochastic signal parameter model to fit stochastic traffic flow patterns. Initially, UAVs and video cameras are used to capture videos of mixed non-motorized traffic flow. The video data were processed with an image detection algorithm based on the YOLO convolutional neural network and a video tracking algorithm using the DeepSORT multi-target tracking model, extracting data on traffic flow, density, speed, and rider characteristics. Then, the autocorrelation and partial autocorrelation functions of the signal are employed to distinguish among four classical stochastic signal parameter models. The model parameters are optimized by minimizing the AIC information criterion to identify the model with optimal fit. The fitted parametric models are analyzed by transforming them from the time domain to the frequency domain, and the power spectrum estimation model is then calculated. The experimental results show that the stochastic capacity model yields a pure EV capacity of 2060–3297 bikes/(h·m) and a pure bicycle capacity of 1538–2460 bikes/(h·m). The density–flow model calculates a pure EV capacity of 2349–2897 bikes/(h·m) and a pure bicycle capacity of 1753–2173 bikes/(h·m). The minimal difference between these estimates validates the effectiveness of the proposed model. These findings hold practical significance in addressing urban road congestion.

## 1. Introduction

Rapid urbanization and economic growth have led to a surge in urban populations and private vehicle numbers, intensifying urban traffic congestion and causing significant inconvenience for urban travel [1,2]. In response, governments worldwide have advocated for low-carbon, environmentally friendly solutions by promoting mixed non-motorized transportation modes. This shift encourages a transition from vehicle-centric travel to more efficient and eco-friendly modes, including public transit, rail systems, and mixed non-motorized vehicles [3,4,5]. Among these modes, mixed non-motorized transportation stands out as environmentally friendly, health-promoting, and convenient, helping to alleviate urban congestion. They have already emerged as a primary a “last-mile” transportation solution in urban settings [6,7]. Consequently, mixed non-motorized vehicles have become integral to urban transportation systems, enhancing urban traffic conditions and serving as a strategic focus in modern urban transportation development.

Currently, severe urban traffic congestion in China has led to increased focus on the role of mixed non-motorized vehicles and pedestrians within integrated urban transportation systems. A comprehensive understanding of mixed non-motorized traffic flow characteristics and capacity within the urban system is needed. China’s mixed non-motorized traffic primarily comprises human-powered and electric bicycles, with significant differences in their traffic flow characteristics. Their combined traffic flow characteristics are more complex than those observed in single-mode traffic [8,9,10]. In this study, mixed non-motorized traffic flow refers specifically to the traffic flow of human-powered and electric bicycles on bicycle lanes. Examining bicycle lane capacity holds both theoretical and practical significance for optimizing urban transportation, improving traffic efficiency, enhancing spatial planning, and bettering the travel experience of urban residents.

This article’s main contribution is the proposal of a stochastic capacity estimation model based on power spectrum estimation, which simulates traffic flow characteristic parameters as stochastic signals and fits a stochastic signal parameter model, providing an improved representation of mixed non-motorized traffic flow dynamics. Additionally, changes in mixed non-motorized traffic flow parameters are analyzed in the frequency domain. The power spectrum estimation model is incorporated to estimate the non-motorized roadway capacity, revealing intrinsic relationships between spectral characteristics and traffic flow parameters. Finally, the collected data on stochastic passing capacity are used to verify the proposed model.

## 2. Literature Review

### 2.1. Study on Mixed Non-Motorized Traffic Flow Characteristics

This study investigates non-motorized traffic flow characteristics and mixed non-motorized lane capacity estimation, selecting three non-motorized lanes on main roads in Nanning and Hangzhou for data collection. A stochastic capacity estimation model based on power spectrum analysis is proposed. Therefore, exploring mixed non-motorized traffic flow characteristics, lane capacity, and passenger car equivalent metrics is necessary. Qin et al. [11] evaluated traffic efficiency concerning the speed characteristics of mixed non-motorized traffic flow. Their simulation of speed distribution and behavioral dynamics within mixed non-motorized traffic flow offers insights for urban management design. Hu et al. [12] examined speed characteristics of non-motorized traffic flow on a G310 national highway section in Henan Province, China, finding a close match between free-flow conditions and ideal speed distributions. Additionally, they identified an exponential relationship between flow rate and density and a cubic relationship between speed and distance. Chen et al. [13] employed mixed-model simulation to analyze traffic performance across different types of non-motorized vehicles under varying density conditions, aiming to optimize traffic efficiency in mixed-road settings. Alexander Bigazzi et al. [14] investigated the impact of electric bicycles on the capacity within bike lanes and mixed traffic settings, introducing the “Bicycle Equivalent Unit” (BEU) concept to measure space occupancy of electric bicycles relative to conventional bicycles, revealing that non-motorized traffic flow exhibits compressibility. Kieran Kalair et al. [15] analyzed high-resolution traffic data fluctuations on a bivariate probability density–flow plane and proposed an innovative real-time anomaly detection method. They found that electric bicycle speeds and flow rates are lower in non-congested conditions compared to congested ones.

In studying mixed non-motorized vehicle traffic flow characteristics, Jin et al. [16] proposed an improved Extended Burgers Cellular Automaton (EBCA) model for heterogeneous bicycle traffic flow, incorporating lane width-related compression probability. This model addresses limitations found in traditional models under various lane widths. Georgios Grigoropoulos [17] quantified traffic flow characteristics such as speed, acceleration, and queue density in mixed non-motorized traffic at major signalized intersections, using the overtaking frequency as an indicator of vehicle interaction, which he found impacts traffic flow efficiency. Maria J et al. [18] introduced a distance-based speed function in a multi-class model to evaluate mixed non-motorized traffic flow, enabling travel time estimation and an overall road performance assessment. Feng et al. [19] examined the extended characteristics of left-turning non-motorized vehicles at intersections, establishing a multiple regression model to explore the relationship between extension degree and related factors, providing theoretical support for optimizing intersection traffic organization. Liu et al. [20] proposed a multi-agent model integrated with deep learning algorithms to simulate traffic conflicts between motor and non-motorized vehicles, accurately predicting evasive maneuvers and collision mechanisms with a strong correlation to actual trajectories.

Further advancements include Gu et al. [21], who developed a Dynamic Correlation Graph Convolutional Network (DCGCN) to construct adjacency matrices from input data using correlation coefficients. Li et al. [22] addressed parameter selection issues for bidirectional long short-term memory (BiLSTM) and kernel extreme learning machine (KELM) models, proposing SHO-BiLSTM and NOA-KELM, respectively, to enhance performance. Huan et al. [23] presented a genetic timing scheduling model (GTSM) for urban traffic signal control, using cellular automata to update timing cycles at multiple intersections, while Wen et al. [24] proposed an inner–outer cellular automaton combined with particle swarm optimization (IOCA-PSO) for real-time urban traffic light optimization.

### 2.2. Study on the Capacity of Mixed Non-Motorized Traffic

In studying bicycle capacity, Dilay Çelebi [25] proposed an integrated approach to optimize bicycle-sharing systems, combining location decision-making with capacity allocation to address unmet demand and balance system use. Navin [26] investigated the relationship between bicycle lane capacity and lane width in Canada, noting that a 2.5 m wide bicycle lane can accommodate up to 10,000 bicycles per hour. Converted to a 1 m wide lane, the capacity is approximately 4000 bicycles per hour. Botma [27] determined that a 1 m wide bicycle lane in the Netherlands has a capacity of 3200 bicycles per hour. Liu et al. [28] conducted a survey and found that Beijing’s average bicycle crossing capacity is approximately 1600 bicycles per hour. Guo et al. [29] conducted field experiments to analyze bicycle traffic dynamics at bottlenecks, finding that capacity remained relatively stable after bottleneck activation. Matthew Brown et al. [30] analyzed GPS data and found that enhanced connectivity and physically separated infrastructure significantly increased bicycle capacity.

### 2.3. Passenger Car Equivalents (PCEs)

When analyzing traffic flows with varied vehicle types, it is essential to compare them on a standardized scale. In this context, traffic volumes of standard cars and various vehicle types are converted into passenger car equivalents (PCEs), an essential method in traffic analysis [31,32]. PCE denotes the number of standard cars equivalent to one vehicle of a specific type in terms of the traffic capacity.

Significant progress has been made in PCE study, resulting in various calculation methods. Classic PCE calculation methods include direct methods (e.g., mathematical models, capacity calculations, speed flow calculations), indirect methods (e.g., overtaking rate, delay calculation, headway calculation, leader vehicle method), and simulation methods [33,34].

Extensive studies have been conducted on conversion factors for electric bicycles relative to conventional bicycles. Given the limitations of existing PCE models in capturing the density characteristics of mixed non-motorized traffic, Zhang et al. [35] proposed a method to evaluate electric bicycle drive system efficiency, establishing a basis for conversion factors between electric and conventional bicycles. Wang et al. [36] developed a model to calculate the conversion factor using video analysis, yielding a factor of 1.3. Jaclyn S [37] analyzed the impact of electric bicycles on traffic flow and speed, finding a PCE of 1.5 for electric bicycles in multimodal traffic, thus highlighting their greater efficiency in high-traffic settings compared to conventional bicycles. These studies indicate that conversion factors for electric bicycles relative to conventional bicycles converge around similar values.

### 2.4. Summary of Study Status

In summary, research on mixed non-motorized traffic flow characteristics, lane capacity, and passenger car equivalents (PCEs) offers theoretical support for the management, design, and evaluation of urban mixed non-motorized lanes. The current research can be summarized as follows:

(1) Current literature on mixed non-motorized traffic flow characteristics primarily describes parameters such as flow, density, and speed on urban roads, using mathematical methods like probability statistics for simulation and analysis.
(2) Studies on mixed non-motorized traffic capacity indicate that foreign researchers primarily use survey data and questionnaires to analyze lane capacity and service level, considering factors like lane width and capacity influences at intersections.

Domestic researchers focus on defining non-motorized lane capacity, mixed traffic flow capacity, lane geometry, and the impact of roadside facilities on capacity. Study methods include metacellular automata simulations and traffic conflict analysis, as well as studies examining conflicts between mixed non-motorized vehicles and passengers near bus stops.

Different road types (e.g., urban roads vs. highways), traffic density, and traffic composition influence PCE calculations. Vehicle type, weight, speed, traffic mobility, and environmental factors should also be considered in PCE calculations.

Existing studies also have some shortcomings:

(1) The stochastic nature of traffic flow changes limits the accuracy of methods like probability statistics, necessitating more complex models for precise description. Furthermore, modeling mixed non-motorized traffic flow often requires idealizing the operational environment, which fails to fully capture traffic flow dynamics.
(2) The current study primarily examines intersections or road sections, often focusing on external interference impacts on mixed non-motorized vehicle flow while neglecting stochastically triggered traffic conflicts during travel.

## 3. Modeling and Methodology

### 3.1. Data Acquisition Methods

#### 3.1.1. YOLO-Based Image Detection

**Figure 1:**
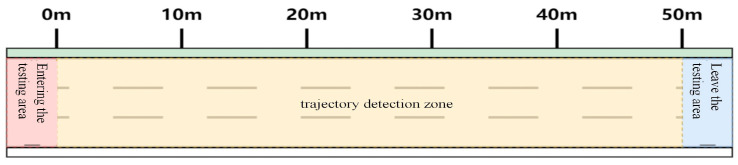
Schematic diagram of detection area.

Detection area setup in aerial video: After aerial video collection, the detection area is defined in the video frame, with specific entrance and exit zones designated. When a vehicle enters the detection entrance, it is identified and assigned a unique number; upon exiting, its identification is removed. The detection area setup is illustrated in Figure 1.

The YOLO (You Only Look Once) algorithm [38] is employed for vehicle recognition in the captured video. YOLO’s convolutional neural network (backbone network) divides the input image into equally sized cells without overlap. Each cell simultaneously participates in convolutional feature extraction, detecting the centroid of each feature layer within the cell. The model then outputs the probability of the target category’s presence and the target’s predicted coordinates at that location [39]. In this study, YOLO is applied to the captured video to detect vehicles in each frame. The model identifies vehicles based on feature frame outputs, including centroid position, length, width, and other relevant data, enabling accurate identification of mixed non-motorized vehicles.

#### 3.1.2. Mixed Non-Motorized Multi-Target Tracking

The DeepSORT multi-target tracking algorithm is utilized for tracking purposes. This tracking algorithm analyzes and extracts trajectory characteristics to address limitations of target detection, effectively removing false detections, enhancing accuracy, and supporting further behavioral analysis [40].

Target tracking involves locating specific targets across video frames, divided into two algorithmic stages. The first stage predicts the positions of targets in the Sth frame, projecting their appearance in the S + Nth frame (candidate region). The second stage verifies if the candidate region in the S + Nth frame corresponds to the initial targets in the Sth frame, initiating tracking if confirmed [41]. The process operates as follows: upon obtaining target detection results, the Kalman filter is initialized with the target frame. An eight-dimensional space represents the state of the track, depicting center position, aspect ratio, target frame height, and corresponding velocity information in image coordinates. This information is used to calculate positional and appearance feature relationships between the prediction frame and target detection frame, enabling association judgment between detection and tracking frames to complete multi-target tracking [42].

#### 3.1.3. Virtual Coil Method

The virtual coil method is used to count traffic captured by the road test camera. At selected road survey points, two parallel lines are demarcated perpendicular to non-motorized road traffic, spaced 4 m apart, with the area between them designated as the virtual coil. This clamped region serves as the traffic video data detection area.

#### 3.1.4. Artificial Assistance Method

The survey revealed that manual assistance is required to determine motor vehicle riders’ gender and age. The questionnaire survey was conducted near the video data collection area, targeting riders passing through the study section. Video recording and the survey were conducted simultaneously.

### 3.2. Data Cleaning

#### 3.2.1. Coordinate Conversion

UAV data collection at high altitudes is influenced by airflow, causing uniform rotational distortion in video frames. This study addresses the issue by repositioning the data origin to the center of the frame and applying coordinate rotation equations (Equations (1) and (2)) for data correction.

(1) $$X_{j}=\sqrt{X_{i}^{2}+Y_{i}^{2}}\cdot \cos(\arctan(\frac{Y_{i}}{X_{i}})-\theta )$$

(2) $$Y_{j}=\sqrt{X_{i}^{2}+Y_{i}^{2}}\cdot \sin(\arctan(\frac{Y_{i}}{X_{i}})-\theta )$$

where $\left(X_{j},Y_{j}\right)$ and $\left(X_{i},Y_{i}\right)$ denote the coordinate values before and after the center rotation process, respectively; *θ* denotes the angle of uniform rotation of this UAV per unit time.

#### 3.2.2. Data Smoothing and Noise Reduction

During video recording, various factors can interfere with recognition and tracking, resulting in outliers in the extracted parameters of mixed non-motorized traffic flow. To address this, an exponential moving average is applied to perform three rounds of smoothing and noise reduction on traffic flow speed and acceleration. This process is described by Equations (3)–(5) as follows:

(3) $$S_{1}(t)=\eta X(t)+(1-\eta )\cdot S_{1}(t-1)$$

(4) $$S_{2}(t)=\eta S_{1}(t)+(1-\eta )\cdot S_{2}(t\cdot 1)$$

(5) $$S_{3}(t)=\eta S_{2}(t)+(1-\eta )\cdot S_{3}(t-1)$$

where $X_{\left(t\right)}$ denotes the actual position of the object at moment *t*; $S_{i}(t)$ denotes the *i*th exponential smoothing value at moment *t*; $S_{i}(t-1)$ denotes the *i*th exponential smoothing value at moment *t* − 1; and *η* denotes the smoothing coefficient (generally taken as 0.6–0.9).

### 3.3. Traffic Flow Speed Distribution Model

#### 3.3.1. Normal Distribution

The normal distribution model is commonly used to describe the operating speed of motorized vehicles and mixed non-motorized traffic flow. Its probability density function is represented by Equation (6):

(6) $$f(x)=\frac{1}{\sqrt{2\pi }\sigma }e^{-\frac{{\left(x-\mu \right)}^{2}}{2\sigma^{2}}}$$

where μ represents the average speed of the mixed non-motorized traffic, and σ denotes the standard deviation of the traffic speed.

#### 3.3.2. Log-Normal Distribution

A stochastic variable follows a log-normal distribution if its logarithm follows a normal distribution. The probability density function of the log-normal distribution is represented by Equation (7):

(7) $$f(x)=\frac{1}{x\sqrt{2\pi }\sigma }e\left(-\frac{{\left(\mathrm{lnx}-\mu \right)}^{2}}{2\sigma^{2}}\right)$$

where μ is the mean speed of the mixed non-motorized traffic, and σ is the standard deviation of the traffic speed.

#### 3.3.3. Gamma Distribution

The gamma distribution is a continuous extension of the Poisson distribution defined over positive real numbers. Its probability density function is represented by Equation (8):

(8) $$f\left(x,\beta ,\alpha \right)=\frac{\beta^{\alpha }}{\Gamma \left(\alpha \right)}x^{\alpha -1}e^{-\beta x},\;x\geq 0$$

where α is the shape parameter, β is the scale parameter, and $\Gamma \left(\alpha \right)$ is the gamma function, which is given by Equation (9):

(9) $$\Gamma (a)=\int_{0}^{+\infty }t^{\alpha -1}e^{-t}dt$$

#### 3.3.4. Weibull Distribution

The Weibull distribution is a continuous probability distribution, with its probability density function defined in Equation (10):

(10) $$f(x)=\left\{\begin{array}{ll} \frac{\eta }{\lambda }\left(\frac{x}{\lambda }\right)e^{-{\left(x/2\right)}^{n}}, & x\geq 0\\ 0, & x<0 \end{array}\right.$$

where λ>0 is the scale parameter, and η>0 is the shape parameter.

When describing speed, it is necessary to determine whether the speed distribution follows a specific distribution model. The Kolmogorov–Smirnov (K-S) test is commonly used for this purpose. The K-S test is a non-parametric method based on the cumulative distribution function. It compares the observed and expected frequencies to determine whether the sample probability distribution conforms to a specified theoretical distribution. To test whether data with *n* observations follow a known distribution $F_{0}\left(x\right)$, assuming the probability distribution of the observed data is $F\left(x\right)$, the formula is given by Equation (11).

(11) $$F\left(x\right)=\frac{N}{n}$$

where N is the number of $x_{i}\leq x$.

According to the Glivenko–Cantelli theorem, when n→∞ occurs, the sample probability distribution ${\hat{F}}_{n}\left(x\right)$ converges uniformly to the population distribution $F\left(x\right)$ with probability 1. Therefore, the distance from $F\left(x\right)$ to $F_{0}\left(x\right)$ can be defined by Equation (12):

(12) $$D=\sup\left|F\left(x\right)-F_{0}\left(x\right)\right|$$

where $\sup\left(\cdot \right)$ is the maximum value function.

The magnitude of *D* is used to evaluate the goodness of fit to the population distribution $F_{0}\left(x\right)$. In the calculation process, if there are n observations, the statistic $D_{n}$ can be used in place of D, and the expression for $D_{n}$ is given by Equation (13):

(13) $$D_{n}=\underset{1\leq i\leq n}{\max}\left\{\max\left(\left|F\left(x_{i}\right)-F_{0}\left(x_{i}\right)\right|,\left|F\left(x_{i-1}\right)-F_{0}\left(x_{i-1}\right)\right|\right)\right\}$$

According to the reference table, when the sample size exceeds 35, the critical value of *D* at a significance level of 0.05 is given by Equation (14):

(14) $$D_{Critical}=\frac{1.36}{\sqrt{n}}$$

where n is the sample size.

If $D_{n}$ exceeds the critical value, it indicates that the sample does not follow the specified known distribution $F_{0}\left(x\right)$; otherwise, it follows the specified distribution.

### 3.4. Stochastic Capacity Estimation Model

The fitting of the stochastic signal $x\left(n\right)$ can be represented using a system function model with a rational function. The expression is given by Equation (15):

(15) $$x\left(n\right)=\overset{q}{\underset{i=0}{\sum }}b_{i}\omega \left(n-1\right)-\overset{p}{\underset{k=1}{\sum }}a_{k}x\left(n-k\right)$$

where $x\left(n\right)$ represents the studied stochastic signal, which, in this model, refers to an array of discrete traffic flow characteristic parameter observations arranged in chronological order; $\omega \left(n\right)$ represents a white noise sequence with a mean of 0 and a variance denoted as $\sigma_{\omega }^{2}$; $\omega \left(n\right)$ needs to be solved during the stochastic signal solution process and can also be understood as the residual of the model during the corresponding order fitting process; and $\sigma_{\omega }^{2}$ serves as the criterion for model fitting in this study and must be determined through actual computation. If it is constant in the final model calculation, it can be proven that the input signal can be described by the fitted model; $b_{l}$ and $a_{k}$ are real constants, while $a_{0}=1$ $b_{l}$, and $a_{k}$ are parameters of multiple regular signals decomposed during stochastic signal fitting. k and l are the orders selected in the stochastic signal simulation model solution process, which can also be understood as the superposition of k or l regular signals.

In practice, appropriate parameter models are selected based on the numerical characteristics of the collected data. Stochastic signals can be approximately represented by an autoregressive model, moving average model, autoregressive moving average model, or differencing integrated moving average autoregressive model.

#### 3.4.1. Autoregressive Model

If a stochastic signal $\left\{x_{n}\right\}$ can be fitted into a model with the structure shown in Equation (16), it is called a bth-order autoregressive model (AR model, autoregressive model), abbreviated as $AR\left(p\right)$:

(16) $$\left\{\begin{array}{l} x(n)=a_{0}+a_{1}x(n-1)+a_{2}x(n-2)+\cdots +a_{p}x(n-p)+\omega (n)\\ a_{p}\neq 0\\ E[\omega (n)]=0,\;Var[\omega (n)]=\sigma_{p}^{2},\;E[\omega (n)\omega (s)]=0,\;s\neq n\\ E[x(n)x(s)]=0,\;\forall s<n \end{array}\right.$$

where $x\left(n\right)$ is the stochastic signal to be analyzed, which represents the collected discrete traffic flow data arranged in chronological order; $a_{1},a_{2},\cdots ,a_{p}$ is a real constant; $a_{p}\neq 0$; $\left\{\omega_{n}\right\}$ is a stationary white noise sequence with zero mean and variance $\sigma_{\omega }^{2}$, which can also be interpreted as the residual between the fitted model and the actual observed values; and $a_{1},a_{2},\cdots ,a_{p}$ and $\sigma_{\omega }^{2}$ are the parameters to be estimated by the model.

#### 3.4.2. Moving Average Model

If the stochastic signal $\left\{x_{n}\right\}$ is fitted into a model with the structure shown in Equation (17), it is called a qth-order moving average model (MA model, moving average model), abbreviated as $MA\left(q\right)$:

(17) $$\left\{\begin{array}{l} x(n)=\mu +\omega (n)-b_{1}\omega (n-1)-b_{2}\omega (n-2)-\cdots -b_{q}\omega (n-q)\\ b_{q}\neq 0\\ E[\omega (n)]=0,Var[\omega (n)]=\sigma_{aa}^{2},E[\omega (n)\omega (s)]=0,s\neq n \end{array}\right.$$

where $x\left(n\right)$ is the stochastic signal to be analyzed, $b_{1},b_{2},\cdots ,b_{q}$ is a real constant, and $b_{q}\neq 0$; $\left\{\omega_{n}\right\}$ is a stationary white noise sequence with zero mean and variance $\sigma_{\omega }^{2}$, representing the residual between the fitted model and the actual observed values. $\mu =\frac{\alpha_{0}}{1-\alpha_{1}-\cdots -\alpha_{p}}$ is a constant; $b_{1},b_{2},\cdots ,b_{q}$ and $\sigma_{\omega }^{2}$ are the parameters to be estimated by the model.

#### 3.4.3. Autoregressive Moving Average Model

If the fitting form of the stochastic signal $\left\{x_{n}\right\}$ follows the structure shown in Equation (18), it is called an Autoregressive Moving Average model (ARMA model), abbreviated as $ARMA\left(p,q\right)$.

(18) $$\;\left\{\begin{array}{l} x(n)=a_{0}+a_{1}x(n-1)+\cdots +a_{p}x(n-p)+\\ \omega (n)-b_{1}\omega (n-1)-\cdots -b_{q}\omega (n-q)\\ a_{p}\neq 0,b_{q}\neq 0\\ E[\omega (n)]=0,Var[\omega (n)]=\sigma_{\omega }^{2},E[\omega (n)\omega (s)]=0,s\neq n\\ E[x(s)\omega (n)]=0,\forall s<n \end{array}\right.$$

where $x\left(n\right)$ is the stochastic signal to be analyzed, $a_{1},a_{2},\cdots ,a_{p}$ and $b_{1},b_{2},\cdots ,b_{q}$ are real constants, $a_{p}\neq 0$, $b_{q}\neq 0$; $\left\{\omega_{n}\right\}$ is a stationary white noise sequence with zero mean and variance $\sigma_{\omega }^{2}$; and $a_{1},a_{2},\cdots ,a_{p}$, $b_{1},b_{2},\cdots ,b_{q}$, and $\sigma_{\omega }^{2}$ are the parameters to be estimated by the model.

The ARMA model combines both the AR model and the MA model, and in parameter estimation, it is necessary to estimate both $a_{1},a_{2},\cdots ,a_{p}$ and $b_{1},b_{2},\cdots ,b_{q}$ simultaneously.

#### 3.4.4. Differencing Integrated Moving Average Autoregressive Model

The ARMA model is one of the key methods in parameter estimation, offering higher accuracy in signal fitting than the AR and MA models. However, AR, MA, and ARMA models are limited to fitting stationary signals. In practice, most collected signals are non-stationary, necessitating an extension of the ARMA model to represent non-stationary stochastic signals. This extension is known as the Autoregressive Integrated Moving Average (ARIMA) model. The ARIMA model incorporates differencing of any order with the ARMA model to describe non-stationary stochastic signals.

### 3.5. Power Spectrum Estimation Model

After applying the Z-transform to $x(n)=\sum_{i=0}^{p}b_{i}\omega (n-1)-\sum_{k=1}^{p}a_{k}x(n-k)$, the expression becomes Equation (19).

(19) $$\sum_{k=1}^{p}a_{k}x(z)z^{-k}=\sum_{i=0}^{p}b_{i}\omega (z)z^{-1}$$

Let $A(z)=\sum_{k=1}^{p}a_{k}z^{-k}$ and $B(z)=\sum_{l=0}^{q}b_{l}z^{-1}$.

Then, the transfer function of the stochastic signal $x\left(n\right)$ after the Z-transform is given by Equation (20):

(20) $$H\left(z\right)=\frac{X\left(z\right)}{W\left(z\right)}=\frac{\sum_{l=0}^{q}b_{l}z^{-1}}{\sum_{k=1}^{p}a_{k}z^{-k}}≜\frac{B\left(z\right)}{A\left(z\right)}$$

The power spectral density $P_{xx}\left(z\right)$ of the parametric model is given by Equation (21):

(21) $$p_{\omega }(z)=\sigma_{\omega }^{2}H(z)H(z^{-1})=\sigma_{\omega }^{2}\frac{B(z)\cdot B(z^{-1})}{A(z)\cdot A(z^{-1})}$$

## 4. Experiments

### 4.1. Data Collection

Traffic flow characterization relies on accurate data; this study focuses on mixed non-motorized traffic flows, which have more complex operational characteristics than motorized traffic flows. The variety of vehicle types, sizes, and speeds on urban non-motorized lanes, along with the need for clear delineation from motorized lanes, introduces more stochasticness into mixed non-motorized traffic flows, complicating data collection. Thus, designing a scientifically sound survey program is essential, including selecting appropriate survey locations, acquiring relevant data types, and pre-processing the data to analyze mixed non-motorized traffic flows.

In this study, only the road width differs between Nanning and Hangzhou, while other road conditions are consistent; three types of non-motorized roads are selected for investigation, with UAVs primarily used to collect traffic flow video data. All survey locations are over 50 m from intersections, bus stops, pedestrian crossings, entrances, and other disturbances, with good road alignment and no slopes, curves, or unusual road forms. Additionally, non-motorized lanes are physically separated from motorized lanes by guardrails, ensuring that mixed non-motorized traffic is unaffected by other factors [43].

Due to varying road conditions across cities, this study adopts two data collection methods. In Nanning, UAVs capture footage, with the YOLO image detection algorithm identifying mixed non-motorized vehicles in the video, and the DeepSORT multi-target tracking algorithm tracking targets and calculates traffic flow data. In Hangzhou, roadside cameras were used for data collection, and the virtual coil method was applied for traffic counting.

In this study, the extracted mixed non-motorized vehicle data fields from the multi-source video include the number, entry and exit times in the detection zone, time difference, vehicle speed (km/h), type, gender, age, and remarks. Type, gender, and age were coded as follows: type (1 for human-powered bicycles, 2 for electric bicycles), gender (1 for males, 0 for females), and age (<29 for young people, 30–49 for middle-aged, and ≥50 for older adults). The basic information for each surveyed road is provided in Table 1.

**Figure 2:**
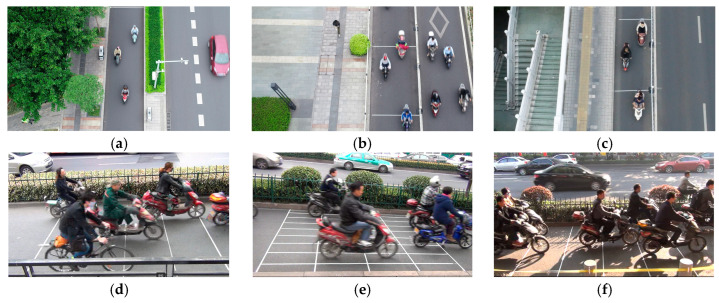
Live view of surveyed roadway. (**a**) Road section A. (**b**) Road section B. (**c**) Road section C. (**d**) Road section D. (**e**) Road section E. (**f**) Road section F.

Figure 2 presents an actual map of each surveyed road section. The surveyed area includes flexible and non-rigid separation facilities, preventing motor vehicle intrusion and influence. The road surface is smooth, with clear markings, ensuring optimal visual conditions for drivers.

Table 2 presents the age and gender statistics of cyclists collected through the questionnaire near each section, while Table 3 provides age and gender statistics obtained through video analysis.

Calculating the error in age and gender ratios using the Euclidean distance shows that the difference between the two methods is relatively small, all within 0.1, indicating that age and gender data obtained via video analysis are reliable. The questionnaire and video analysis results overlap, making both methods valuable for cross-referencing. Part of the error may arise because the questionnaire survey is a sampling method, meaning respondents near the surveyed section may not fully represent all non-motorized cyclists on the road.

### 4.2. Traffic Flow Characteristics Analysis

#### 4.2.1. Statistical Characteristics Analysis

A statistical analysis of traffic flow data uses observational data to examine the essential characteristics and underlying traffic flow patterns. This is a fundamental method for studying traffic flow issues. An analysis of mixed traffic flow characteristics includes factors such as types of mixed non-motorized vehicles, rider characteristics, traffic flow volume, and vehicle speed [44].

In this study, video image detection and object tracking algorithms are used to obtain video parameters of mixed non-motorized vehicles. The time when each non-motorized vehicle enters the detection area is recorded as $t_{1}$, and the time when it leaves the detection area is recorded as $t_{2}$. The length of the detection area is *L* = 15 m, and the speed of the non-motorized vehicle is denoted as v, which is calculated using Equation (22).

(22) $$\nu =\frac{L}{t_{2}-t_{1}}\;$$

In the equation, *v* represents the speed of the non-motorized vehicle; $t_{1}$ and $t_{2}$ are the times when the vehicle enters and leaves the detection area, respectively; and L is the length of the detection area.

Because non-motorized vehicle lanes lack strict lane divisions, area density is used to represent mixed non-motorized vehicle density. Defined as the number of mixed non-motorized vehicles per unit area at a given time, it is denoted by *K*, with the formula provided in Equation (23):

(23) $$k=\frac{N}{L\times W}=\frac{N}{L\times (W-0.5)}$$

In the equation, *N* is the number of mixed non-motorized vehicles within the observation range at a given time; *L* is the observation length; *w* is the effective width of the non-motorized vehicle lane; and *W* is the width of the non-motorized vehicle lane.

Study indicates that varying sampling time intervals can significantly impact the predictions of non-motorized vehicle lane capacity [45]. Given the short saturation time of mixed non-motorized traffic flow, a 30 s sampling interval was used. Based on the literature review in Section 2, which summarizes current research on passenger car equivalents, this study uses a conversion factor of 1.34 for electric bicycles relative to traditional bicycles, which was derived from previous research.

A statistical description of the survey data is provided in Table 4.

Table 4 shows that traffic volume in the surveyed sections is relatively high, providing a sufficient sample size. A small number of drivers were observed traveling in the wrong direction. Due to the low incidence of wrong-way driving, its impact is considered minimal in this study.

**Figure 3:**
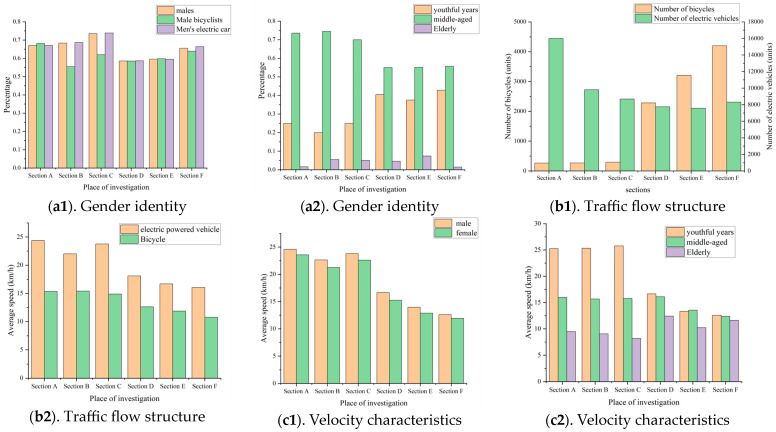
Characteristics of traffic flow statistics on the investigated roadway. (**a1**). Gender identity. (**a2**). Gender identity. (**b1**). Traffic flow structure. (**b2**). Traffic flow structure. (**c1**). Velocity characteristics. (**c2**). Velocity characteristics.

Figure 3 presents the rider characteristics by gender, age, and vehicle type. In Figure 3a, the average proportion of male riders across the six surveyed sections is significantly higher than that of female riders, with males accounting for approximately 60%. This suggests male riders are more likely to use mixed non-motorized vehicles and may have a higher demand for speed, making them more inclined to choose electric bicycles over traditional bicycles. Figure 3b shows that most mixed non-motorized vehicle riders are middle-aged, though there are notable differences in age distribution between the two cities. In Nanning, riders aged 0–29 constitute 23.28%, lower than 42.85% in Hangzhou, suggesting regional differences in mixed non-motorized traffic flow. In Nanning’s three surveyed sections, mixed non-motorized vehicle proportions are 98.36%, 97.33%, and 96.74%, while in Hangzhou, they are 77.28%, 70.26%, and 66.44%. Figure 3c also highlights significant differences in mixed non-motorized vehicle proportions between the two cities, suggesting that regional culture and economic development shape mixed non-motorized traffic flow characteristics. In summary, the analysis methods for mixed non-motorized traffic flow should be tailored to each city, underscoring the need to study the characteristics of emerging mixed non-motorized traffic flows as examined in this paper.

Figure 3 compares the average speeds of mixed non-motorized vehicles across six sections by vehicle type, rider gender, and rider age. The significant variation in average speeds across different types of mixed non-motorized vehicles is primarily due to performance differences between electric and traditional bicycles. Additionally, male and middle-aged riders exhibit higher average speeds across all sections, consistent with prior study findings [46]. Moreover, average speeds between the two cities vary significantly across age groups, influenced by regional culture, transportation infrastructure, and management policies.

#### 4.2.2. Traffic Flow Speed Distribution Analysis

**Figure 4:**
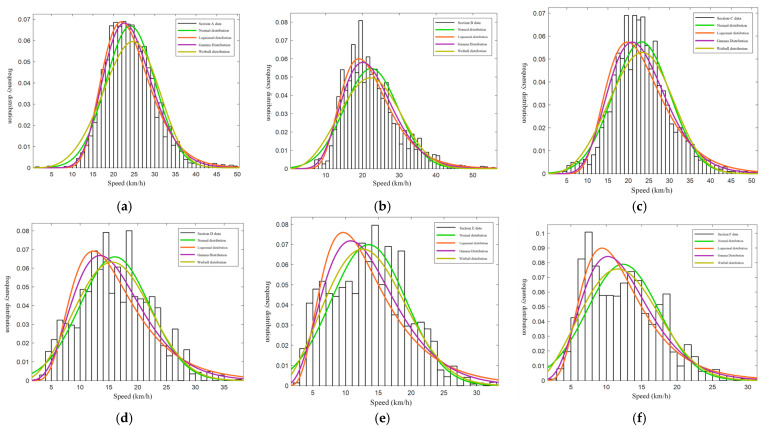
Speed distribution of surveyed road sections. (**a**) Segment A speed profiles. (**b**) Segment B speed profiles. (**c**) Segment C speed profiles. (**d**) Segment D speed profiles. (**e**) Segment E speed profiles. (**f**) Segment F speed profiles.

Existing studies typically describe the probability distribution of mixed non-motorized vehicle operating speeds using a normal distribution. However, given the complexity of mixed non-motorized traffic flows and varying speed distributions across sections, researchers suggest using log-normal, gamma, and Weibull distributions to better represent these speeds [47]. Based on current findings, this study applies normal, log-normal, gamma, and Weibull models to describe the operating speeds of mixed non-motorized traffic across sections. These models fit and test the speed frequency distribution for each section, with results shown in Figure 4.

As shown in the speed distribution fitting diagram, the speed distribution of mixed non-motorized vehicles is dispersed due to significant speed differences among various vehicle types. This leads to interference and frequent conflicts between non-motorized vehicles, significantly impacting safety. For example, electric vehicles can reach speeds up to 56 km/h, exceeding the national limit of 25 km/h and posing a hidden risk to traffic safety.

The K-S test indicates a relatively poor fit for the distribution model when applied to mixed speeds of each road section. This suggests that mixed non-motorized vehicle flow speeds are influenced by multiple factors, adding stochasticness and complexity to their distribution characteristics. Traditional single-distribution models cannot adequately describe the speed distribution of mixed non-motorized vehicles.

#### 4.2.3. Stochastic Passability Estimation Based on Power Spectrum Estimation

**Figure 5:**
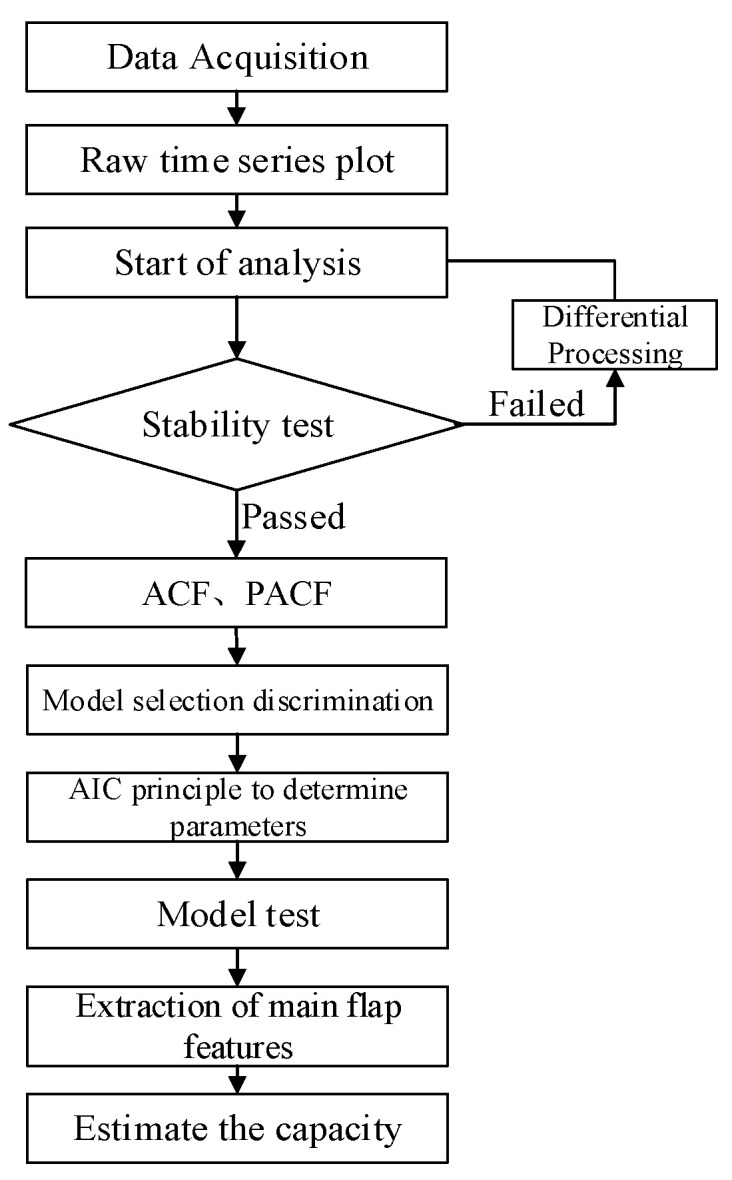
Flowchart for modeling the stochastic capacity model.

The power spectrum estimation model reveals intrinsic patterns of stochastic signal variation in the frequency domain. The central petal in the power spectrum represents the highest peak, reflecting the intensity of the dominant frequency component, obtained after stochastic factors are eliminated. This peak, or central petal, indicates the passing capacity. The stochastic passing capacity model, based on power spectrum estimation, follows the steps shown in Figure 5:

Step 1: Smoothness analysis: Extract traffic flow data as a time-varying stochastic signal, and test the signal for smoothness to verify if the time series is stationary;

Step 2: Differencing: If the plotting and ADF tests indicate the time series is non-stationary, apply differencing to obtain a stationary sequence;

Step 3: Model identification: Determine model parameters based on autocorrelation and partial autocorrelation properties to select an appropriate range;

Step 4: Model optimization: Use the AIC and BIC criteria to find the optimal parameter combination and test for statistical significance;

Step 5: Model testing: Conduct hypothesis testing to determine if the sequence is white noise and evaluate model superiority;

Step 6: Calculate passing capacity: Extract the main peak intensity to determine the passing capacity of the road section.

This study selected the following road sections for experiments, with their primary conditions listed in Table 5. After data preprocessing, 2846 traffic flow observations were collected, using a 30 s statistical interval. The collected traffic flow video captures high-density traffic, including free-flowing conditions and severely congested periods. These three road sections were selected for stochastic signal analysis, allowing for the examination of each traffic state as it traverses. This analysis can reveal the underlying patterns of traffic flow characteristics within each road section.

**Figure 6:**
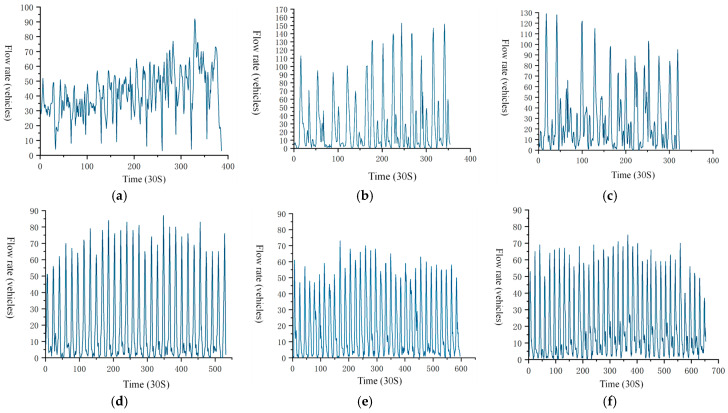
Time series of flow on surveyed road sections. (**a**) Roadway A Flow Timing Chart. (**b**) Roadway B Flow Timing Chart. (**c**) Roadway C Flow Timing Chart. (**d**) Roadway D Flow Timing Chart. (**e**) Roadway E Flow Timing Chart. (**f**) Roadway F Flow Timing Chart.

After processing the traffic flow characteristics of the selected road sections, the traffic flow rate was plotted in Figure 6, using the sampling interval as a reference. Figure 6 illustrates the relationship between time and traffic flow rate across the surveyed sections. Each increment on the time axis represents a 30 s interval.

The stationarity of the signal can be preliminarily observed in Figure 6. The figure shows that the flow time series curve for section D fluctuates around a horizontal line, indicating that the mean of the flow time series signal is relatively stable, with minimal changes in the amplitude over time. This suggests that the variance is also stable, indicating that the flow stochastic signal for section D can be considered generally stationary. Similarly, the speed time series graphs for sections E and F also suggest stationary signals. Further testing is needed for signals without an observable trend in the time series graph. The primary method for stationarity testing is the unit root test, which examines whether a unit root exists in the sequence. If a unit root is present, the sequence is non-stationary; if it is absent, the sequence is stationary. Stationarity test results are presented in Table 6.

Table 6 presents the test statistic and corresponding *p*-value for each data collection, calculated through the stationarity test. The null hypothesis assumes the presence of a unit root, indicating a non-stationary time series. By testing signal stationarity and comparing probability values with significance levels of 1%, 5%, and 10%, it was found that the null hypothesis is not rejected for flow rates in sections A and B, indicating non-stationarity. The flow rates in sections C and F, with test statistics of −2.811 and −2.843 and *p*-values of 0.057 and 0.052, respectively, are below the 10% significance level, indicating stationary signals. The flow rate in section D, with a *p*-value of 0.035 at the 5% significance level, rejects the null hypothesis, indicating stationarity. The flow rate in section E, with a *p*-value of 0.001 at the 1% significance level, also rejects the null hypothesis, confirming stationarity. To meet stationarity requirements for power spectrum modeling, differencing must be applied to non-stationary sequences.

After first-order differencing on initially non-stationary signals, stationarity testing confirmed that the signals became stationary.

**Figure 7:**
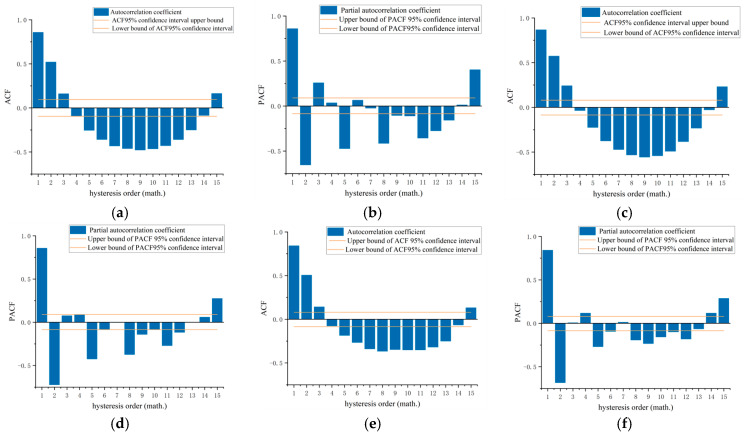
Images of flow signals on surveyed roadway segments. (**a**) Autocorrelation images of road segment C flows. (**b**) Segment A flow-biased autocorrelation image. (**c**) Autocorrelation images of road segment B flows. (**d**) Segment B flow biased autocorrelation image. (**e**) Autocorrelation images of road segment C flows. (**f**) Segment C flow biased autocorrelation image.

To reduce the computational load for the stochastic capacity model, this study performed autocorrelation and partial autocorrelation analyses to preliminarily determine the p and q orders. Figure 7 presents the autocorrelation and partial autocorrelation function graphs for traffic flow signals in sections A, B, and C.

In the power spectrum estimation model, the autocorrelation and partial autocorrelation function graphs exhibit two characteristics: tailing and cut-off. These characteristics can be used for preliminary testing to identify the presence of p-lags or q-lags. Figure 7 shows a clear geometric decay in the autocorrelation functions of the three sections, indicating p-lags in their flow signals. The partial autocorrelation function graphs for the three sections consistently show non-zero values, suggesting q-lags in all three signals. By analyzing the autocorrelation and partial autocorrelation functions of the selected section flow signals within a 95% confidence interval, the *p*- and q-values for the power spectrum estimation model were determined, as shown in Table 7. Here, d represents the model’s differencing order.

The AIC information criterion was used to further refine the power spectrum model parameters and obtain optimal values. The residuals of the fitted model were tested using Q-statistic values, followed by a goodness-of-fit test. Using road section A as an example, Table 8 presents the flow model test results for section A.

Table 8 shows that the optimal parameters for the flow model of road section A, based on the AIC criterion, are ARIMA (3, 1, 2). Additionally, the optimal models for each section are as follows: road section B—ARIMA (2, 1, 3); road section C—ARIMA (2, 0, 3); road section D—ARIMA (2, 0, 4); road section E—ARIMA (2, 0, 2); and road section F—ARIMA (2, 0, 2).

**Figure 8:**
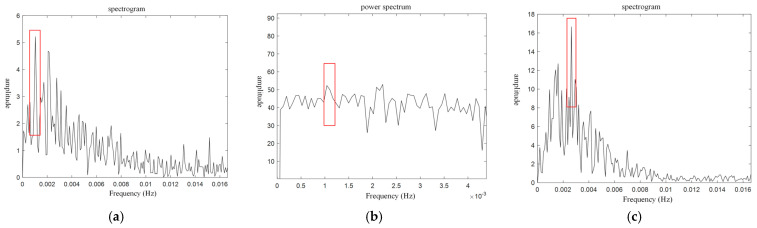

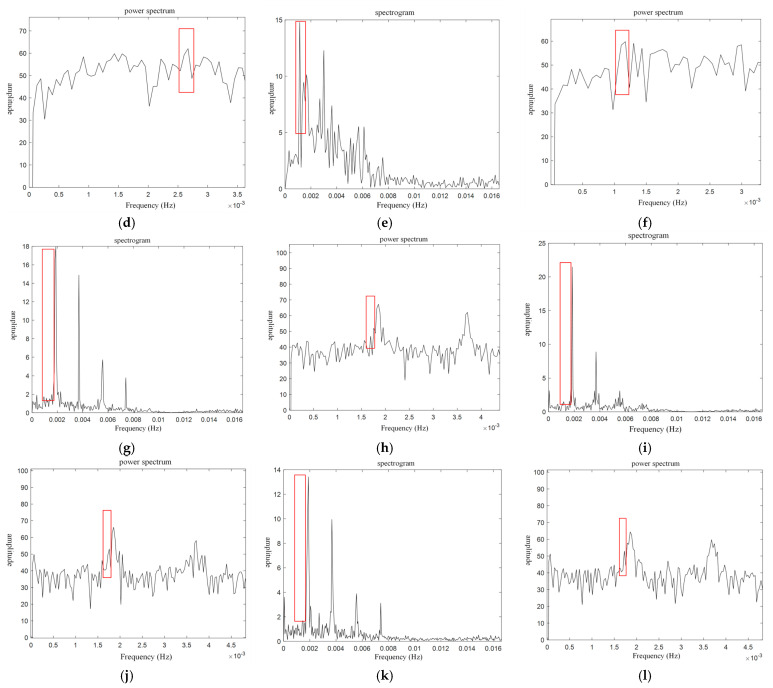
Survey roadway flow map. (**a**) Flow spectrogram of section A. (**b**) Flow power spectra of section A. (**c**) Flow spectrogram of section B. (**d**) Flow power spectra of section B. (**e**) Flow spectrogram of section C. (**f**) Flow power spectra of section C. (**g**) Flow spectrogram of section D. (**h**) Flow power spectra of section D. (**i**) Flow spectrogram of section E. (**j**) Flow power spectra of section E. (**k**) Flow spectrogram of section F. (**l**) Flow power spectra of section F.

Figure 8 presents the power and frequency spectra of traffic flow for the selected section. In the frequency spectrum, the component with the highest intensity is marked by a red box, representing the signal with the greatest amplitude variation derived from the stochastic signal. The corresponding frequency in the power spectrum, also highlighted by a red box, represents the capacity sought by this model, indicated by the peak value of the most prominent component.

In the power spectrum model, the main peak represents the highest point in the graph, and its intensity reflects the maximum stable flow rate for the road. In this study, the intensity of the main peak in the power spectrum is converted to non-motorized road flow per unit time and unit width, representing the non-motorized road capacity targeted in this analysis.

From the power spectrum image, the primary eigenvalues were extracted, as shown in Table 9.

To calculate capacity, traffic flow is uniformly converted to the flow of mixed non-motorized vehicles per unit time and unit width, expressed in bikes/(h·m). The calculation formula is provided in Equation (24).

(24) $$Q=\frac{q}{n\times w}\times 3600=\frac{q}{n\times (W-0.5)}\times 3600$$

In the equation, q represents the number of mixed non-motorized vehicles passing within the statistical interval; q is the primary lobe intensity value obtained from the power spectrum model; n refers to the time interval, which in this paper is set to 30 s, w represents the effective width of the non-motorized vehicle lane; and W refers to the total width of the non-motorized vehicle lane.

The traffic flows examined in this study underwent vehicle equivalent calculations within the stochastic capacity estimation model based on power spectrum analysis. Accordingly, the capacity estimates for the selected road sections were converted to equivalent pure EV and bicycle capacities, as shown in Table 10.

**Figure 9:**
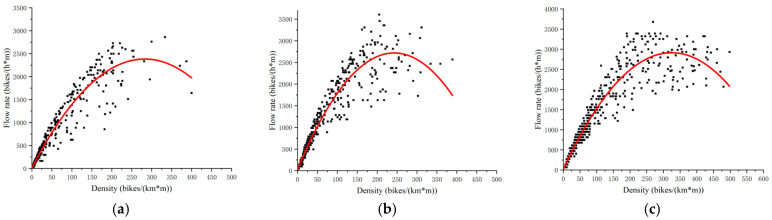
Density–flow map of surveyed roadway segments. (**a**) Density–flow diagram for section D. (**b**) Density–flow diagram for section E. (**c**) Density–flow diagram for section F.

To validate the effectiveness of the proposed traffic capacity estimation model, a comparison was made with existing density–flow relationship models. Figure 9 shows the scatter plots and fitting curves for the density–flow relationship in non-motorized lanes of sections D, E, and F. Observing the scatter plots reveals that the overall trend of the points follows a power function form. After comparing several power functions, the quadratic function was found to provide the best fit, so it was selected to model the flow-density distribution. The fitted density–flow models and calculation results for each section are presented in Table 11.

The comparison shows that the stochastic capacity model developed in this study aligns closely with the results of the density–flow model. This validates the stochastic capacity model, further enriches the theory of mixed non-motorized traffic flow, and provides theoretical support for non-motorized lane management.

## 5. Conclusions

This study focuses on mixed non-motorized vehicles on urban road sections, investigating traffic flow characteristics and capacity in non-motorized lanes. The main study conclusions are as follows:

(1) UAVs and cameras were used to collect video data on non-motorized lanes with hard segregation on urban roads, capturing mixed non-motorized traffic flow characteristics. Preliminary analyses were conducted on rider and vehicle characteristics. Speed data were fitted to normal, lognormal, gamma, and Weibull distributions, demonstrating the stochasticness and volatility of mixed traffic flow.
(2) A stochastic capacity model was constructed based on power spectrum estimation. By analyzing stochastic signal characteristics, this study introduces the frequency domain concept to investigate changes in traffic flow characteristics. Using power spectrum estimation, the stochastic signal model is fitted to traffic flow data, with AR, MA, ARMA, and ARIMA models selected based on autocorrelation and partial autocorrelation functions. Optimal parameters are chosen via the AIC criterion, followed by time–frequency domain transformation. The primary peak intensity in the power spectrum is used to measure passability.
(3) An empirical validation of the stochastic capacity model was conducted. The calculations of the investigated traffic flow data estimate pure electric vehicle (EV) capacity at 2060–3297 bikes/(h·m) and pure bicycle capacity at 1538–2460 bikes/(h·m). The density–flow model estimates place capacity at 2349–2897 bikes/(h·m) for pure electric vehicles and 1753–2173 bikes/(h·m) for pure bicycles. The comparison confirms that the capacity estimates from the stochastic capacity model align with existing studies.

## Figures and Tables

**Table 1:** Basic information of surveyed road sections.

Cities	Sites	Breadth	Abbreviations
Nanning	Minzu Avenue (next to Shimen Forest Park)	3.55 m	Section A
	Minzu Avenue and Qingxiu Road East Section	3 m	Section B
	Yongjiang Bridge Road Section	2.2 m	Section C
Hangzhou	Dongxin-Shaoxing junction, Dongxin road section	3.65 m	Section D
	Hushu South-Chaowang Intersection	2.93 m	Section E
	Teachers-Wen Er Road junction	2.27 m	Section F

**Table 2:** Statistical data table on age and gender for the questionnaire survey.

Cities	Sites	Percentage of Age			Percentage by Gender	
		0–29 Years	30–49 Years	50 Years and Over	Male	Female
Nanning	Minzu Avenue (next to Shimen Forest Park)	24.87%	71.48%	3.65%	72.68%	27.32%
	Minzu Avenue and Qingxiu Road East Section	23.37%	72.79%	3.84%	74.26%	25.74%
	Yongjiang Bridge Road Section	30.35%	66.57%	3.08%	76.41%	23.59%
Hangzhou	Dongxin-Shaoxing junction, Dongxin road section	52.10%	44.72%	3.17%	67.41%	32.59%
	Hushu South-Chaowang Intersection	45.59%	52.16%	2.25%	65.18%	34.82%
	Teachers-Wen Er Road junction	56.97%	40.69%	2.34%	68.17%	31.83%

**Table 3:** Statistical data table on age and gender for video discrimination.

Cities	Sites	Percentage of Age			Percentage by Gender	
		0–29 Years	30–49 Years	50 Years and Over	Male	Female
Nanning	Minzu Avenue (next to Shimen Forest Park)	21.63%	73.51%	4.86%	66.97%	33.03%
	Minzu Avenue and Qingxiu Road East Section	20.50%	74.51%	4.99%	68.31%	31.69%
	Yongjiang Bridge Road Section	25.94%	70.01%	4.05%	73.50%	26.50%
Hangzhou	Dongxin-Shaoxing junction, Dongxin road section	46.52%	48.88%	4.60%	58.52%	41.48%
	Hushu South-Chaowang Intersection	42.21%	55.14%	2.65%	59.57%	40.43%
	Teachers-Wen Er Road junction	53.25%	43.15%	3.60%	65.57%	34.43%

**Table 4:** Statistical description of road section survey data.

Cities	Investigated Road Sections	Lane Width	Sample Size	Percentage of Electric Vehicles	Percentage of Males	Proportion of Youth
Nanning	Minzu Avenue (next to Shimen Forest Park)	3.55 m	16,268	98.36%	66.97%	21.63%
	Minzu Avenue and Qingxiu Road East Section	3 m	10,076	97.33%	68.31%	20.50%
	Yongjiang Bridge Road Section	2.2 m	8979	96.74%	73.50%	25.94%
Hangzhou	Dongxin-Shaoxing junction, Dongxin road section	3.65 m	10,037	77.28%	58.52%	46.52%
	Hushu South-Chaowang Intersection	2.93 m	10,782	70.26%	59.57%	42.21%
	Teachers-Wen Er Road junction	2.27 m	12,515	66.44%	65.57%	53.25%
average value		2.93 m	11,442	84.40%	65.41%	35.01%

**Table 5:** Basic information of selected surveyed road sections.

Cities	Sites	Width	Abbreviations
Nanning	Minzu Avenue (next to Shimen Forest Park)	3.55 m	Section A
	Minzu Avenue and Qingxiu Road East Section	3 m	Section B
	Yongjiang Bridge Road Section	2.2 m	Section C
Hangzhou	Dongxin-Shaoxing junction, Dongxin road section	3.65 m	Section D
	Hushu South-Chaowang Intersection	2.93 m	Section E
	Teachers-Wen Er Road junction	2.27 m	Section F

**Table 6:** Smoothness test table.

Data	Test Statistic	*p*-Value	1%	5%	10%	H
Section A Flow	−2.188	0.027	−3.466	−2.877	−2.575	0
section B Flow	−1.251	0.651	−3.471	2.88	−2.576	0
Section C Flow	−2.811	0.057	−3.47	−2.879	−2.576	1
Section D Flow	−3.002	0.035	−3.474	−2.879	−2.576	1
Section E Flow	−4.941	0.001	−3.465	−2.877	−2.575	1
Section F Flow	−2.843	0.052	−3.472	−2.88	−2.576	1

**Table 7:** Table of parameter ranges for power spectrum estimation.

Data	Model Parameter		
	*p*	d	q
Flow rate of Section A	4	1	4
Flow rate of Section B	4	1	5
Flow rate of section C	3	0	3
Flow rate of section D	4	0	4
Flow rate of section E	4	0	3
Flow rate of section F	4	0	3

**Table 8:** Flow modeling test table for section A.

ARIMA Model (3, 1, 2) Test Table			
Term	Symbol	Value	*p*-Value
Q-Statistics	Q6 (*p*-value)	1.533	0.957
	Q12 (*p*-value)	12.635	0.068
	Q18 (*p*-value)	50.932	0.000
	Q24 (*p*-value)	55.897	0.000
	Q30 (*p*-value)	56.955	0.000
Information criterion	AIC	1927.95	
Goodness of fit	R^2^	0.879	

**Table 9:** Flow power spectrum feature extraction.

Sections	Main Valve Strength	Frequency Values
Section A	52.3595	0.001042
Section B	62.0679	0.002669
Section C	59.8066	0.001172
Section D	62.2261	0.001855
Section E	64.1054	0.001855
Section F	62.3808	0.001855

**Table 10:** Estimated capacity.

Sections	Pure Electric Vehicle Capacity (Bikes/(h·m))	Pure Bicycle Capacity (Bikes/(h·m))
Section A	2060	1538
Section B	2979	2223
Section C	3262	2434
Section D	2370	1769
Section E	3077	2296
Section F	3297	2460

**Table 11:** Density–flow model fit table.

Sections	(Math.) Simultaneous Equations	$R^{2}$	Pure Electric Vehicle Capacity (Bikes/(h·m))	Pure Bicycle Capacity (Bikes/(h·m))
Section A	$Q=-0.0279k^{2}+16.2446k$	0.928	2349	1753
Section B	$Q=-0.04485k^{2}+21.9347k$	0.938	2647	1975
Section C	$Q=-0.02572k^{2}+16.95613k$	0.941	2867	2139
Section D	$Q=-0.0297k^{2}+16.78492k$	0.933	2382	1778
Section E	$Q=-0.04568k^{2}+22.17318k$	0.932	2718	2028
Section F	$Q=-0.02752k^{2}+17.85613k$	0.934	2912	2173

## Data Availability

The datasets presented in this article are not readily available because the data are part of an ongoing study. Requests to access the datasets should be directed to chenjianqiu@unn.edu.cn.
